# Re-repair vs. Replacement for Failed Mitral Valve Repair: A Systemic Review and Meta-Analysis

**DOI:** 10.3389/fcvm.2022.868980

**Published:** 2022-06-14

**Authors:** Zhaoji Zhong, Hang Xu, Wu Song, Sheng Liu

**Affiliations:** Department of Cardiovascular Surgery, Fuwai Hospital, Chinese Academy of Medical Science, Beijing, China

**Keywords:** mitral valve repair, failure, recurrence, reoperation, mitral valve replacement

## Abstract

**Objective:**

The objective of this study was to compare outcomes of re-repair with those of mitral valve replacement (MVR) for failed initial mitral valve repair (MVr).

**Methods:**

We searched the Pubmed, Embase, and Cochrane Library databases for studies that compared mitral valve re-repair with MVR for the treatment of failed initial MVr. Data were extracted by two independent investigators and subjected to a meta-analysis. Odds ratio (OR), risk ratio (RR), hazard ratio (HR), ratio difference (RD), mean difference (MD), and 95% confidence interval (CI) were calculated with the Mantel-Haenszel and inverse-variance methods for mode of repair failure, perioperative outcomes, and follow-up outcomes.

**Results:**

Eight retrospective cohort studies were included, with a total of 938 patients, and mean/median follow-up ranged from 1.8 to 8.9 years. Pooled incidence of technical failure was 41% (RD: 0.41; 95% CI: 0.32 to 0.5; *P* = 0.00; I^2^ = 86%; 6 studies, 846 patients). Pooled mitral valve re-repair rate was 36% (RD: 0.36; 95% CI: 0.26–0.46; *P* = 0; I^2^ = 91%; 8 studies, 938 patients). Pooled data showed significantly lower perioperative mortality (RR: 0.22; 95% CI: 07 to 0.66; I^2^ = 0%; *P* = 0.008; 6 studies, 824 patients) and significantly lower long-term mortality (HR:0.42; 95% CI: 0.3 to 0.58; I^2^ = 0%; *P* = 0; 7 studies, 903 patients) in the re-repair group compared with MVR.

**Conclusions:**

Mitral valve re-repair was associated with better immediate and sustained outcomes for failed MVr and should be recommended if technically feasible.

## Introduction

Mitral regurgitation (MR) is common, with a prevalence of 0.6–2.7% in the general population (1, 2). Yearly mortality rates with medical treatment in patients ≥ 50 years old are approximately 6% for severe organic MR (3). Surgery has been proven to improve symptoms and survival, and mitral valve repair (MVr) is associated with lower operative mortality and better long-term survival than mitral valve replacement (MVR) (4). Thus, MVr is the treatment of choice recommended by current guidelines, especially for degenerative diseases (5, 6).

In experienced heart centers, a repair rate of nearly 100% for degenerative MR can be achieved (7). However, MVr carries a potential risk for reoperation. The reoperation rate following degenerative MVr is approximately 0.5–1% per year, reducing late survival (5, 6, 8, 9). Re-repair, MVR (10–19), and transcatheter procedures (20–22) have been recommended for failed MVr, but controversies exist regarding optimal treatment strategy. Re-repair and MVR are two strategies for reoperation of failed previous MVr. Some reported similar re-repair results compared with MVR (23), while others adopted an aggressive strategy for re-repair (24).

Thus, we conducted this systematic review and meta-analysis to compare the long-term outcomes of re-repair and MVR for failed previous MVr.

## Methods

### Search Strategy

The study was performed according to the Preferred Reporting Items for Systematic Reviews and Meta-Analyses (PRISMA) guidelines (25). On January 14, 2022, a comprehensive literature search was conducted using the PubMed, Embase, and Cochrane Library databases for relevant studies published since 2000 that reported the outcomes of reoperation for failed MVr. The following words found in the title or abstract were used to perform the search: “mitral valve” and “repair” and “recurrent^*^” or “reoperation.”

### Study Selection

Studies that met the following criteria were included: (1) the population consisted of patients who underwent repeat mitral valve surgery (re-repair or MVR) because of failed previous MVr; (2) there were data on perioperative mortality and long-term survival outcomes; (3) studies that reported the outcomes of the re-repair and MVR groups.

The exclusion criteria were as follows: (1) primary surgery including MVR, (2) reoperation due to causes other than repair failure, (3) transcatheter procedures, (4) second cross-clamp during the same surgery, and (5) studies with ≤ 10 patients included, duplicate publications, and review of articles.

Three authors (ZZ, HX, and WS) screened and assessed the studies independently for inclusion. Disagreements regarding the inclusion of articles were resolved *via* group consensus.

### Data Extraction

Two authors (ZZ and HX) reviewed and extracted the reported data from the studies, including details of the study (study design, inclusion criteria, study period, and follow-up duration), baseline demographics, preoperative echocardiographic parameters, causes of repair failure (technique or valve-related), surgical details (re-repair/MVR, and cross-clamp time), perioperative morbidities and mortality, and long-term outcomes (MR recurrence, reoperation, and mortality). For long-term outcomes, the number of observed events and the Kaplan-Meier estimation were extracted and analyzed.

### Quality Assessment

Study quality and risk of bias were assessed using the Newcastle-Ottawa Scale. Disagreements were resolved by consensus.

### Statistical Analysis and Meta-Analysis

The analyses were performed utilizing Review Manager 5.4 (Cochrane Collaboration, Oxford, United Kingdom). Statistical heterogeneity was assessed using I^2^ for low (25~49%), moderate (50~ 74%), and high (≥ 75%) heterogeneity. When I^2^ ≥ 50%, random-effects models were utilized.

For the single-arm meta-analysis (re-repair rate and incidence of technical failure), generic inverse variance methods were used. For binary data (incidence of technical failure), the odds ratio (OR) was calculated using the Mantel-Haenszel method. For continuous data (aortic cross-clamp time and hospital stay time), the mean difference (MD) was calculated using the inverse variance method. For binary data (perioperative and follow-up events), the relative risk (RR) was calculated using the Mantel-Haenszel method. For time-to-event data (the long-term outcomes), the hazard ratio (HR) was calculated using the logarithmic scale generic inverse variance method (26). When HR was not provided, it was calculated using the Kaplan-Meier survival *P* value and other details if possible (26, 27). When the mean and variance were not provided, they were estimated from the median and quartile range (28–30). Forest plots were generated from the present pooled results.

## Results

### Study Selection

**Figure 1:**
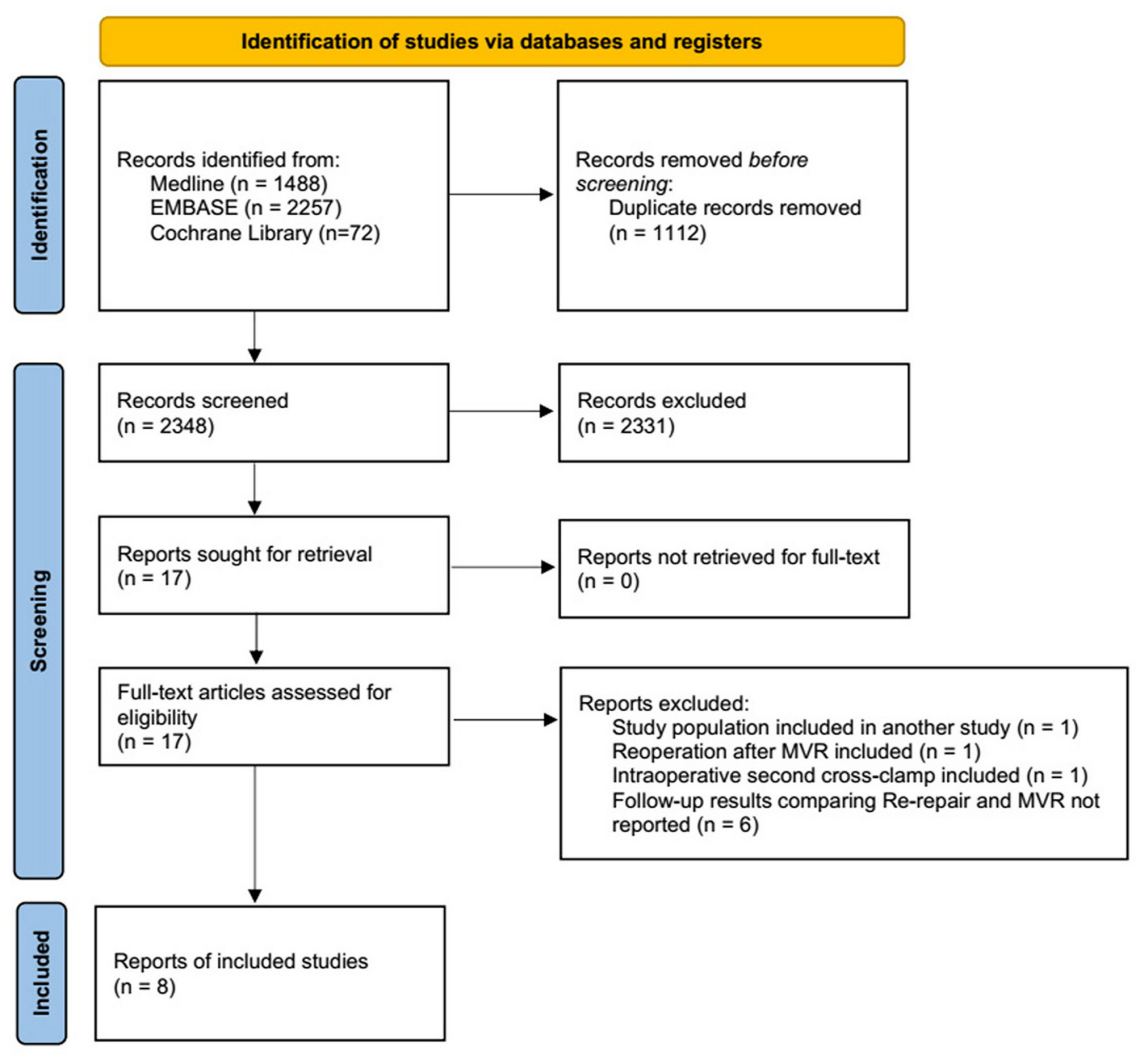
PRISMA flow chart depicting the selection of studies included in the meta-analysis. PRISMA, Preferred Reporting Items for Systematic Reviews and Meta-analyses; MVR, mitral valve replacement.

A total of 2,365 studies were identified using the search criteria. Based on the title and abstract, 17 studies were retrieved for full-text review. Follow-up outcomes comparing re-repair and MVR were not reported in six studies. Two studies by Zegdi et al. used patients from the same institute (AP-HP, Assistance Publique-Hôpitaux de Paris) for two different periods: 1991–2004 (31) and 1987–2006 (14), and the first one was excluded. One study by Ma et al. reported a second cross-clamp under reinstituted cardiopulmonary bypass for incomplete initial repair but not repair failure (32). Kwedar et al. included reoperation after both previous MVr and MVR and did not report outcomes after initial repair specifically (33). The remaining eight studies comprised pooled data (Figure 1).

### Study Characteristics

The eight studies were all unmatched retrospective cohort ones. Four studies included only degenerative mitral regurgitation, while the other four studies did not specify the pathological types. A total of 938 patients were included, of whom 295 underwent re-repair and 643 underwent MVR. Six studies compared the preoperative demographic data between the re-repair and MVR groups. All the studies reported follow-up results; mean/median follow-up ranged from 1.8 to 8.9 years. Seven studies reported the Kaplan-Meier survival estimation of the re-repair and MVR groups, while one only reported the follow-up events. The basic characteristics of the studies are listed in Table 1, and the demographic data are shown in Table 2. The Newcastle-Ottawa Scale scores ranged from 7 to 9 out of 9 for the studies. Some points were lost because of a lack of comparability between the groups. The quality assessment of the included studies is listed in Supplementary Table 1.

**Table 1:** Basic characteristics of the included studies.

	**Study period**	**Country**	**Pathology**	**Patients**		**Follow-up (y)**		**Main conclusions**
				**Re-repair**	**MVR**	**Re-repair**	**MVR**	
Suri et al. (12)	1970–2005	U.S.	Degenerative	64	81	4.1 ± 5.1		Re-repair has superior survival and heart function
Dumont et al. (13)	1980–2005	U.S.	Degenerative	68	120	6.5 ± 5		When performed, re-repair is durable
Zegdi et al. (14)	1987–2006	France	Degenerative	21	22	6.2 (0.83–15.8)	8.9 (0–14.2)	Re-repair is feasible with encouraging results
Nishida et al. (15)	1991–2015	Japan	Degenerative	23	63	6.4 ± 4.6		Re-repair has acceptable durability and survival
Kilic et al. (16)	2002–2014	U.S.	Not specified	48	257	3.9 ± 3.3	3.8 ± 3.1	Re-repair has outcomes comparable to MVR
Noack et al. (18)	1996–2016	Germany	Not specified, Ring dehiscence only	19	38	5.6 ± 4.4		MV reoperation can be performed safely
Trumello et al. (17)	2003–2017	Italy	Not specified	39	40	7.4 ± 3.3		With re-repair, recurrent MR is not rare, but survival tends to be better
El-Eshmawi et al. (19)	2011–2020	U.S.	Not specified, Redo for stenosis only	13	22	1.8(0.5–3.8)		Re-repair is feasible in a select group of patients

*MVR, mitral valve replacement; MV, mitral valve*.

**Table 2:** Baseline demographic data.

	**Age (y)**			**Male (** ***n*** **, %)**			**Preoperative LVEF (%)**			**Preoperative LVEDD (mm)**		
	**Re-repair**	**MVR**	***P***	**Re-repair**	**MVR**	***P***	**Re-repair**	**MVR**	***P***	**Re-repair**	**MVR**	***P***
Suri et al. (12)	64 ± 13	67 ± 11	0.24	49 (77%)	53 (65%)	0.15	52 ± 12	56 ± 11	0.23	56 ± 7	57 ± 8	0.47
Dumont et al. (13)	57 ± 9.8	63 ± 12	**0.0005**	50 (73%)	69 (57%)	**0.03**	53 ± 8.5	50 ± 10	0.14	-	-	
Zegdi et al. (14)	55 (10–87)	66 (27–78)	>0.05	15 (71%)	17 (77%)	>0.05	71 (45–80)	67 (35–79)	>0.05	-	-	
Nishida et al. (15)	49 ± 2.8	63 ± 1.7	**<0.001**	31 (36%)			56 ± 2.3	55 ± 1.4	0.55	59 ± 8		
Kilic et al. (16)	61 ± 16	62 ± 15	0.96	30 (62%)	135 (45%)	**0.02**	19% <40	15% <40	0.5	-	-	
Noack et al. (18)	60 ± 12			42 (74%)			52 ± 16			-	-	
Trumello et al. (17)	50 ± 13	60 ± 11	**<0.001**	25 (64%)	24 (60%)	0.707	59 (55–62)	57 (51–60)	0.175	56 ± 9	57 ± 8	0.426
El-Eshmawi et al. (19)	61.4 ± 11.4			11 (31%)			58.5 ± 9.3			46 ± 7		

*MVR, mitral valve replacement; LVEF, left ventricular ejection fraction; LVEDD, left ventricular end-diastolic dimension. The bold values indicate significantly difference*.

### Mode of Mitral Valve Repair Failure

Failure of MVr can be categorized into technical failure (procedure-related) or progression of disease (valve-related) for recurrent regurgitation (34). Causes of mitral stenosis after MVr included organic and functional (“tunnel effect” due to a small ring) (19).

The proportion of technical failure was reported in six studies (12–17) and showed high heterogeneity (I^2^ = 86%). One study (18) only included reoperation due to ring dehiscence (technical failure). The pooled incidence of technical failure was 41% (RD: 0.41; 95% CI:0.32–0.5; *P* = 0; I^2^ = 86%; 6 studies, 846 patients).

**Figure 2:**
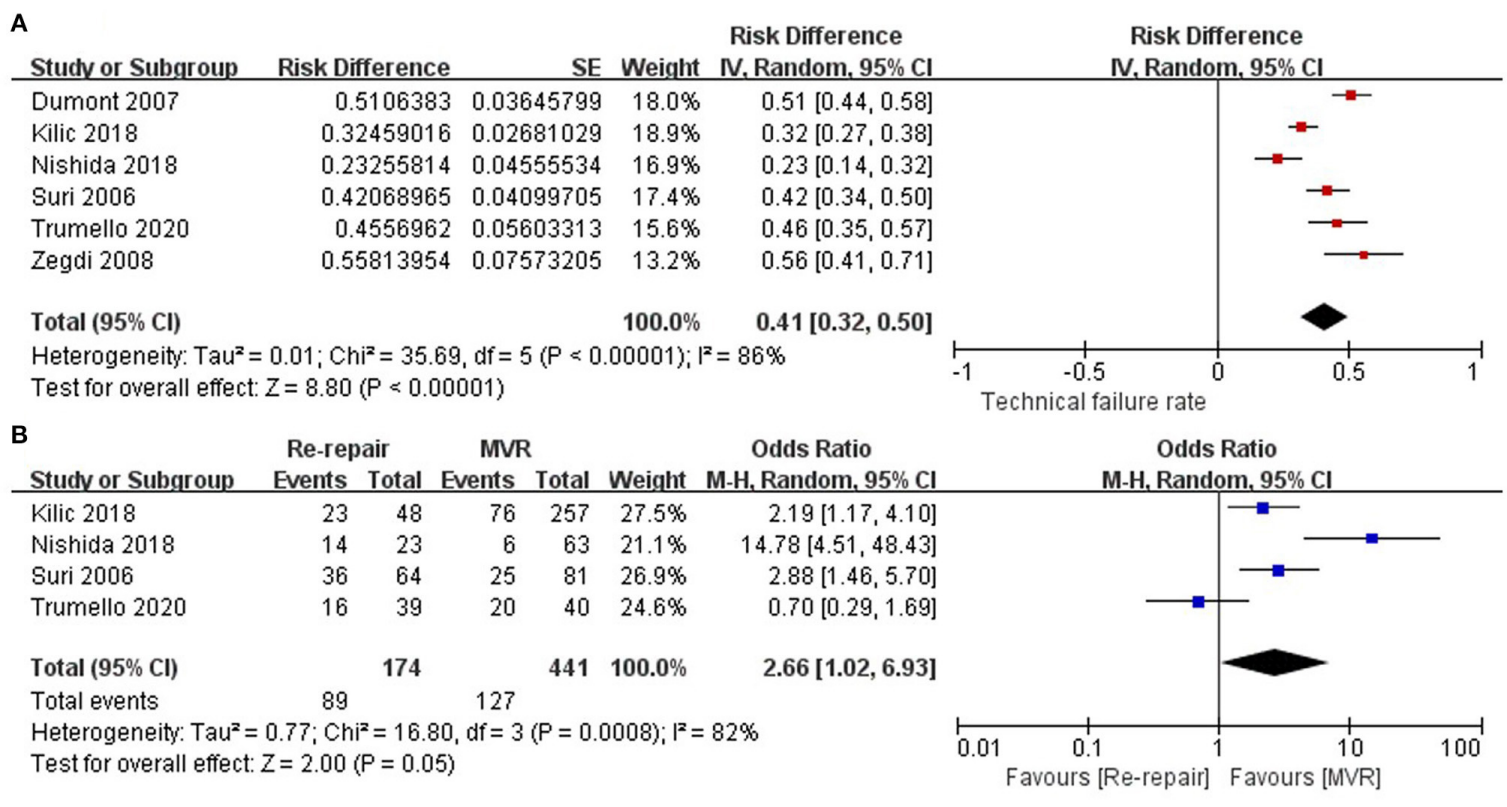
Mode of mitral valve repair failure. **(A)** Incidence of technical failure of the entire cohort; **(B)** incidence of technical failure in the re-repair and MVR groups. SE, standard error; IV, inverse variance; CI, confidence interval; M-H, Mantel-Haenszel; MVR, mitral valve replacement.

The incidence of technical failure in the re-repair and MVR groups was reported in four studies (12, 15–17). The incidence of technical failure was higher in the re-repair group, but the difference was not significant (OR: 2.66; 95% CI: 1.02–6.93; *P* =0.05; I^2^ = 82%; 4 studies, 615 patients) (Figure 2).

### Perioperative Outcomes

The pooled mitral valve re-repair rate was 36% (RD 0.36; 95% CI: 0.26–0.46; *P* = 0; I^2^ = 91%; 8 studies; 938 patients).

Mean aortic cross-clamp time was reported by three studies (15–17) and was shorter in the re-repair group, but the difference was not significant (MD = −11.5 min; 95% CI: −23.03–0.02; *P* =0.05; I^2^ = 82%; 3 studies, 470 patients).

Perioperative mortality was reported in all the studies, including two studies (17, 19) without perioperative death. The pooled data of the other six studies (12–16, 18) showed significantly lower perioperative mortality in the re-repair group than in the MVR group (RR: 0.22; 95% CI:0.07–0.66; I^2^ = 0%; *P* = 0.008; 6 studies, 824 patients).

**Figure 3:**
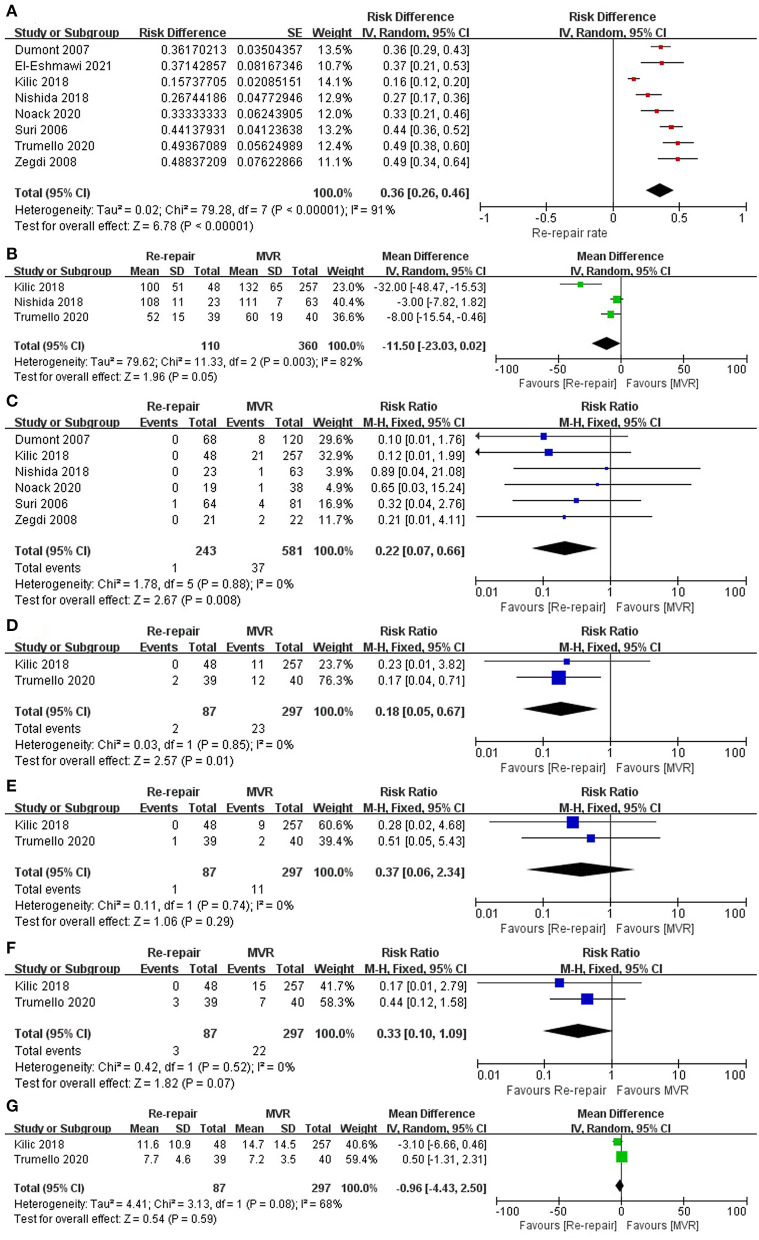
Perioperative outcomes. **(A)** Re-repair rate for the entire cohort; **(B)** aortic cross-clamp time; **(C)** perioperative mortality; **(D)** AKI; **(E)** stroke; **(F)** reoperation for bleeding; **(G)** postoperative hospital stay. SE, standard error; IV, inverse variance; CI, confidence interval; M-H, Mantel-Haenszel; MVR, mitral valve replacement; AKI, acute kidney injury.

Major perioperative morbidities were reported in two studies (16, 17). There was a significant difference in the risk of acute kidney injury (AKI) (RR: 0.18; 95% CI:0.05–0.67; I^2^ = 0%; *P* = 0.01; 2 studies, 384 patients, but not in stroke (RR: 0.37; 95% CI:0.06–2.34; I^2^ = 0%; *P* = 0.29; 2 studies, 384 patients) and reoperation for bleeding (RR: 0.33; 95% CI:0.1–1.09; I^2^ = 0%; *P* = 0.07; 2 studies, 384 patients). There was no significant difference in length of hospital stay (MD = −0.96 d; 95% CI: −4.43–2.5; *P* = 0.59; I^2^ = 68%; 2 studies, 384 patients) (Table 3; Figure 3). Low cardiac output syndrome, blood transfusion, mediastinitis, and other morbidities were not pooled because of heterogeneity in the diagnostic criteria.

**Table 3:** Perioperative outcomes.

	**Tech failure**		**Cross-clamp time (min)**		**Perioperative** **AKI (%)**		**Perioperative** **stroke (%)**		**Reoperation for** **bleeding (%)**		**Perioperative** **Mortality (%)**		**Hospital stays** **(Day)**	
	**Re-repair**	**MVR**	**Re-repair**	**MVR**	**Re-repair**	**MVR**	**Re-repair**	**MVR**	**Re-repair**	**MVR**	**Re-repair**	**MVR**	**Re-repair**	**MVR**
Suri et al. (12)	36 (56%)	25 (31%)	-	-							1 (1.6%)	4 (4.9%)	-	-
Dumont et al. (13)	96 (51%)		-	-	6 (3.2%)		9 (4.8%)		10 (5.3%)		0	8 (6.6%)	-	-
Zegdi et al. (14)	24 (56%)		-	-	0		0		1 (2.3%)		0	2 (9.1%)	11 (5–28)	
Nishida et al. (15)	14 (61%)	6 (10%)	108 ± 11	111 ± 7	2 (2.3%)		3 (3.5%)		-	-	0	1 (1.6%)	-	-
Kilic et al. (16)	23(47.5%)	76 (29.5%)	100 ± 51	132 ± 65	0	11 (4.2%)	0	9 (3.5%)	0	15 (6%)	0	21 (8%)	11.6 ± 10.9	14.7 ± 14.5
Noack et al. (18)	19 (100%)	38 (100%)	59 ± 33		4 (7.0%)		2 (3.5%)		-	-	0	1 (2.6%)	-	-
Trumello et al. (17)	16 (41%)	20 (50%)	52 ± 15	60 ± 19	2 (5.1%)	12 (30%)	1 (2.6%)	2 (5%)	3 (8.1%)	7 (18%)	0	0	7 (5–11)	7 (5–9.5)
El-Eshmawi et al. (19)	-	-	107.2 ± 28.4		0		-	-	-	-	0	0	10 (7–15)	

*MVR, mitral valve replacement; LVEF, left ventricular ejection fraction; LVEDD, left ventricular end-diastolic dimension; AKI, acute kidney injury*.

### Long-Term Outcomes

Follow-up reoperation was reported in six studies (12–16, 19) and was significantly higher in the re-repair group (RR: 2.11; 95% CI: 1.03–4.28; I^2^ = 0%; *P* = 0.04; 6 studies, 802 patients).

**Figure 4:**
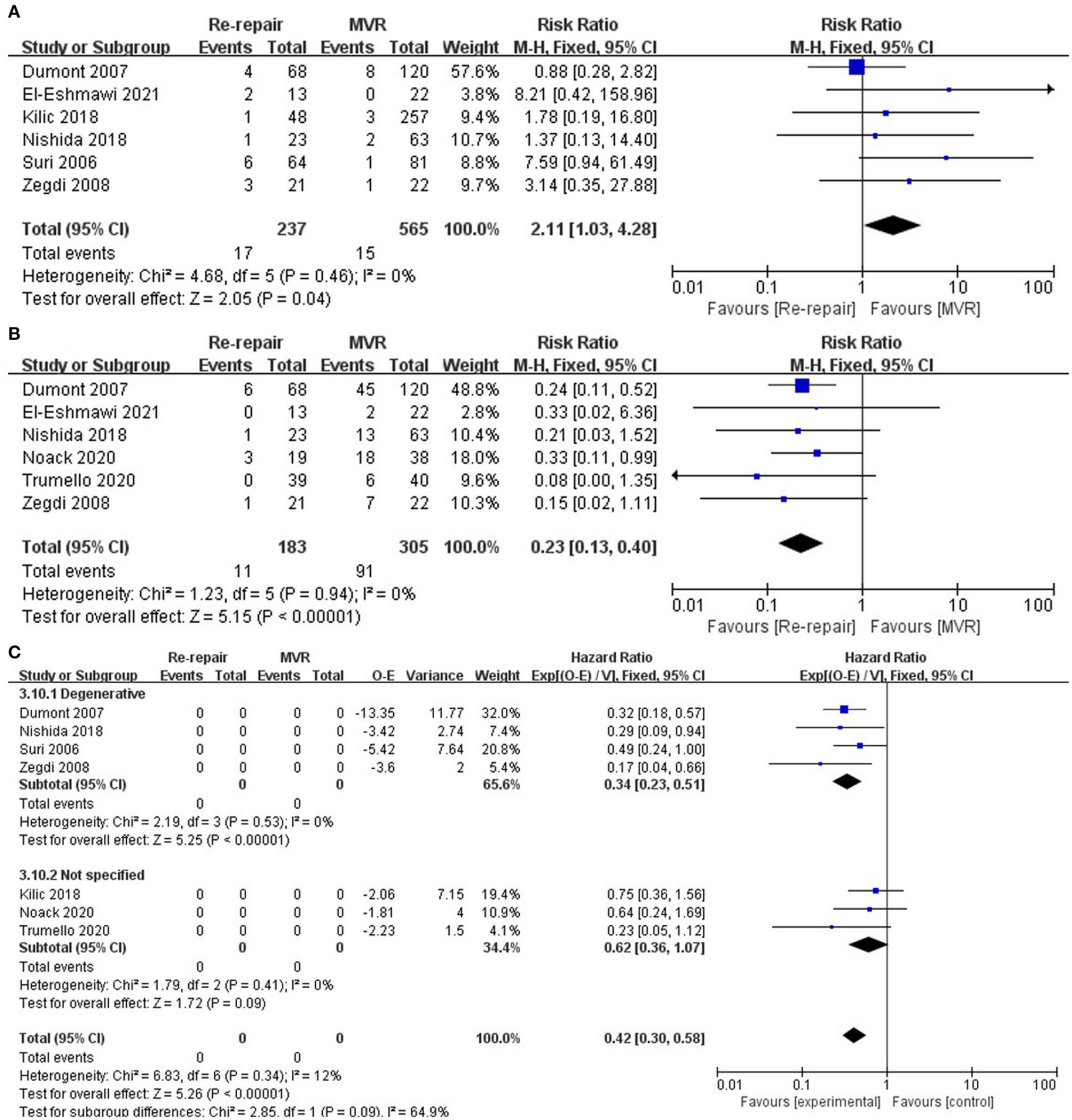
Long-term outcomes. **(A)** Reoperation; **(B)** follow-up death, risk ratio pooled; **(C)** follow-up mortality, hazard ratio pooled. O-E, Observed minus expected events; IV, inverse variance; CI, confidence interval; M-H, Mantel-Haenszel; MVR, mitral valve replacement.

Follow-up death was reported in six studies (13–15, 17–19) and was significantly lower in the re-repair group (RR: 0.23; 95% CI: 0.13–0.4; I^2^ = 0%; *P* = 0; 6 studies, 488 patients). In addition, re-repair was associated with significantly lower long-term mortality when HR was pooled for analysis (HR: 0.42; 95% CI: 0.3–0.58; I^2^ = 0%; *P* = 0; 7 studies, 903 patients). Finally, increase in long-term survival was observed in studies that only reported degenerative diseases (12–15) (HR 0.34; 95% CI: 0.23–0.51; I^2^ = 12%; *P* = 0.00; 4 studies, 462 patients). Pooled data from studies reporting mixed pathological types (16–18) showed lower long-term mortality in the re-repair group but without significant difference (HR: 0.62; 95% CI: 0.36–1.07; I^2^ = 0%; *P* = 0.09; 3 studies, 441 patients) (Table 4; Figure 4).

**Table 4:** Long-term outcomes.

	**MR Recurrence** **≥Moderate**		**Event: Redo (%)**		**Freedom form** **Redo (%)**			**Event: Death (%)**		**Survival (%)**		
	**Re-repair**	**MVR**	**Re-repair**	**MVR**	**Re-repair**	**MVR**	**Significant difference**	**Re-repair**	**MVR**	**Re-repair**	**MVR**	**Significant difference**
Suri et al. (12)	-	-	6 (9.4%)	1 (1.2%)	83%, 5 y	93%, 5y for entire cohort		31 (21.4%)		76%, 5 y	60%, 5 y	**Yes** **(*****P*** **=** **0.05)**
Dumont et al. (13)	-	-	4 (5.9%)	8 (6.7%)	93%, 10 y	87%, 10 y		6 (8.8%)	45 (37.5%)	81%, 12 y	45%, 12y	**Yes** **(*****P*** **<** **0.0001)**
Zegdi et al. (14)	2 (9.5%)	0 (0%)	3 (14.3%)	1 (4.5%)	95%, 7 y	95%, 7 y		1 (4.8%)	7 (31.8%)	95%, 7 y	69%, 7 y	**Yes** **(*****P*** **=** **0.011)**
Nishida et al. (15)	2 (8.7%)	0 (0%)	1 (4.3%)	2 (3.2%)	95.7%, 5 y	98.3%, 5 y	No (log rank = 0.94)	1 (4.3%)	13 (20.6%)	100%, 5y	82%, 5 y	**Yes** **(log rank** **=** **0.039)**
Kilic et al. (16)	-	-	1 (2.1%)	3 (1.2%)						78%, 5 y	68%, 5y	No (*P* = 0.29)
Noack et al. (18)	-	-	-	-	-	-		3 (15.8%)	18 (47.4%)	79.1%, 5 y	70.9%, 5 y	No (*P* = 0.366)
Trumello et al. (17)	29.2%, 8 y	0%, 8 y	0	0	-	-		0	6 (15%)	100%, 8 y	96.5%, 8 y	No (*P* = 0.069)
El-Eshmawi et al. (19)	-	-	2 (15.4%)	0	-	-		0	2 (9.1%)	93.5%, 5y for entire cohort		-

*MVR, mitral valve replacement; MR, mitral regurgitation. The bold values indicate significantly difference*.

## Discussion

In this systematic review and meta-analysis, the major finding was that re-repair for failed previous MVr was associated with significantly improved perioperative and long-term survival. This is the first systematic review and meta-analysis that focused on optimal treatment for failed previous MVr. Veerappan et al. (23) included a second cross-clamp under reinstituted cardiopulmonary bypass (32) and redo after MVR (33).

### Advantages of Re-repair

The pooled data in our meta-analysis suggested that mitral valve re-repair was associated with significantly lower perioperative mortality (*P* = 0.008). For primary surgery, perioperative mortality was significantly lower for MVr than for MVR in both unmatched and matched populations (*P* < 0.001) (4). This was similar to that of reoperative mitral valve surgery. Kwedar et al. analyzed data from Medicare and found that hospital mortality was 9.8% for re-repair, 12.7% for MVR with bioprosthesis, and 12.2% for MVR with mechanical prosthesis (33). Re-repair has lower perioperative morbidity (16, 17). Relatively simple techniques and preservation of the subvalvular apparatus might contribute to the improved short-term outcomes.

Mitral valve re-repair significantly improved late survival in our meta-analysis (RR and HR, *P* = 0) despite the higher risk of a third reoperation (*P* = 0.04). Even in institutes with a “very low threshold for re-repair” and high re-repair rate, re-repair has excellent survival and durability (24). A previous meta-analysis found similar long-term survival between re-repair and MVR, but a second cross-clamp under reinstituted cardiopulmonary bypass was included in the study (23). In a subgroup analysis of studies that did not specify the pathology, the long-term benefit of re-repair was not significant (*P* = 0.09). However, Aphra et al. reported that patients with rheumatic mitral valve disease showed excellent survival after re-repair, although there was a certain risk of failure over time (35). In addition, patients who underwent re-repair had significantly better ejection fractions and smaller LV end-systolic dimensions during follow-up (12).

Thus, mitral valve re-repair was associated with better immediate and sustained outcomes and should be recommended if technically feasible.

### Feasibility of Re-repair

The pooled mitral valve re-repair rate of the studies was 36% (95% CI 0.26–0.46), ranging from 16 to 49%. Anyanwu et al. reported a systemic strategy to re-repair in all cases, and the re-repair rate was 85% (90% for degenerative disease) (24). Even mitral stenosis after MVr could be re-repaired in an experienced mitral valve reference center (19). Several factors can influence the decision to re-repair. When analyzing the mode of repair failure and valve structure, the surgeon should keep in mind that both early and long-term survival would be improved if durable re-repair could be achieved.

In studies that reported re-repair procedures in detail (15, 17, 24, 35), the most frequently used techniques include leaflet resection, ring removal/annuloplasty, edge-to-edge repair, and neochordae. Simple techniques such as edge-to-edge repair may be an effective choice (36). The technique should be selected based on individual mitral valve lesions, while the basic principles and steps of MVr outlined by Carpentier should always be adhered to.

It is not clear what kind of repair failure is suitable for re-repair with long-term durability. Our meta-analysis suggested that the percentage of technical failure might be higher in the re-repair group. This suggests that re-repair should be considered if there is a definite lesion in the previously repaired location.

### Implications for Percutaneous Transcatheter Procedures

Percutaneous transcatheter procedures have been increasingly used for failed MVr in selected patients, including mitral clips (20), neochords (21), and valve-in-rings (22). However, the current literature is limited by the small number of patients and short follow-up period. There is a paucity of data on the long-term outcomes of these interventional procedures. Our meta-analysis suggested that transcatheter MVr might be chosen over transcatheter mitral valve replacement.

### Limitations

Our meta-analysis has several limitations. First, there were only limited studies comparing the long-term survival of re-repair and MVR. The included studies were retrospective and observational, with relatively small sample sizes. Second, there was significant heterogeneity among the included studies regarding the patients' baseline characteristics and outcomes. Third, there was no uniform standard for selecting re-repair or MVR, and the surgical approach was selected based on the experience of the surgeon or institution. Fourth, most of the studies included in the meta-analysis focused on reintervention and survival, and few studies reported on the follow-up functional status and echocardiographic evaluation. Finally, the studies included mainly patients with degenerative mitral regurgitation. The optimal treatment for rheumatic and infective disease has yet to be studied.

## Conclusions

Mitral valve re-repair should be recommended for failed MVr if technically feasible, because re-repair has improved perioperative and long-term results compared with MVR.

## Data Availability Statement

The original contributions presented in the study are included in the article/Supplementary Material, further inquiries can be directed to the corresponding author.

## Author Contributions

ZZ and SL conceived and designed the study. ZZ, HX, and WS collected and analyzed the data and wrote the manuscript. ZZ, HX, WS, and SL reviewed and edited the manuscript. All authors contributed to the article and approved the submitted version.

## Funding

This study was funded by the Capital Science and Technology Program, Beijing (Grant no: Z201100005520005).

## Conflict of Interest

The authors declare that the research was conducted in the absence of any commercial or financial relationships that could be construed as a potential conflict of interest.

## Publisher's Note

All claims expressed in this article are solely those of the authors and do not necessarily represent those of their affiliated organizations, or those of the publisher, the editors and the reviewers. Any product that may be evaluated in this article, or claim that may be made by its manufacturer, is not guaranteed or endorsed by the publisher.
