# Supplementary material for: Re-repair vs. Replacement for Failed Mitral Valve Repair: A Systemic Review and Meta-Analysis
Source: Front Cardiovasc Med. 2022 Jun 14;9:868980. doi: 10.3389/fcvm.2022.868980 (PMC9237322; doi:10.3389/fcvm.2022.868980)
Supplement: Supplementary file 1 [file Table_1.DOCX]

**Supplementary Table 1. Quality assessment of included studies using the Newcastle-Ottawa Scale**

|  | **Selection** | | | | **Comparability** | | **Outcome** | | | **Overall** |
| --- | --- | --- | --- | --- | --- | --- | --- | --- | --- | --- |
|  | **Representativeness of exposed cohort** | **Selection of the nonexposed cohort** | **Ascertainment of exposure** | **Demonstration that outcome of interest was not present at start of study** | **Comparability of cohorts on the basis of the design or analysis (LVEF)** | **study controls for any additional factor (Age)** | **Assessment of outcome** | **Was follow-up long enough for outcomes to occur (>1.5y)** | **Adequacy of follow up of cohorts** |  |
| **Suri 2006** | 1 | 1 | 1 | 1 | 1 | 1 | 1 | 1 | 1 | 9 |
| **Dumont 2007** | 1 | 1 | 1 | 1 | 1 | 0 | 1 | 1 | 1 | 8 |
| **Zegdi 2008** | 1 | 1 | 1 | 1 | 1 | 1 | 1 | 1 | 1 | 9 |
| **Nishida 2018** | 1 | 1 | 1 | 1 | 1 | 0 | 1 | 1 | 1 | 8 |
| **Kilic 2018** | 1 | 1 | 1 | 1 | 1 | 1 | 1 | 1 | 1 | 9 |
| **Noack 2020** | 1 | 1 | 1 | 1 | 0 | 0 | 1 | 1 | 1 | 7 |
| **Trumello 2020** | 1 | 1 | 1 | 1 | 1 | 0 | 1 | 1 | 1 | 8 |
| **El-Eshmawi 2021** | 1 | 1 | 1 | 1 | 0 | 0 | 1 | 1 | 1 | 7 |

LVEF: left ventricular ejection fraction
